# Three-Dimensional Computed Tomography–Based Mapping of the Intraosseous Safe Zone for Acetabular Cup Screw Fixation: A Retrospective, Single-Center Cohort Study

**DOI:** 10.2106/JBJS.OA.26.00322

**Published:** 2026-10-27

**Authors:** Takamitsu Sato, Rui Hirasawa, Shigeo Hagiwara, Hiroyuki Kaneko, Hiroyuki Hamano, Yuya Kawarai, Junichi Nakamura, Seiji Ohtori

**Affiliations:** 1 Department of Orthopedic Surgery, Graduate School of Medicine, Chiba University, Chiba, Japan; 2 Japan Data Science Consortium Co., Ltd, Bunkyo-ku, Tokyo, Japan

## Abstract

**Background:**

Conventional safe zones are defined by the proximity of acetabular cup screws to intrapelvic vessels; however, this relationship varies among individuals. This study aimed to define the intraosseous safe zone in which the screws remain entirely within bone without penetrating the medial wall.

**Methods:**

Three-dimensional computed tomography data for 30 hips of female patients with normal acetabular morphology were analyzed. A hemispherical cup was positioned virtually, and screw trajectories were evaluated in 24 directions using a clock-face coordinate system referenced to the ipsilateral anterior superior iliac spine. Bone thickness was measured in each direction, and intraosseous safe zones were defined for screw lengths of 15, 20, 25, 30, 40, and 50 mm. Heatmaps were generated to visualize the distribution of safe regions, and the angular range in which screws remained intraosseous in all cases was determined.

**Results:**

The median patient age was 50.1 years. The intraosseous safe zone was consistently located in the posterosuperior region for all screw lengths. The extent of the safe zone significantly decreased as the screw length increased (p < 0.01), whereas shorter screws expanded the safe zone toward the posteroinferior region. The angular range of the intraosseous safe zone was consistently the greatest at the 11 and 11:30 o’clock positions for 15, 20, and 25 mm screws.

**Conclusions:**

The findings provide a useful framework for preoperative planning and conceptualizing safe screw trajectories in total hip arthroplasty.

**Clinical Relevance:**

Directing screws toward the posterosuperior acetabulum, particularly around the 11 o’clock region, may enhance safety by maintaining intraosseous fixation and avoiding intrapelvic neurovascular injury. Shorter screws may expand safe fixation options toward the posteroinferior region.

## Introduction

In cementless total hip arthroplasty (THA), using acetabular cup screws near major intrapelvic vessels introduces the risk of iatrogenic vascular injury, which can be limb-threatening or life-threatening^1,2^. Vascular injuries occur in approximately 0.04% of primary THA procedures, increasing to approximately 0.19% in revision cases, with mortality rates of approximately 7%, amputation rates of approximately 1%-2%, and persistent ischemia in approximately 7% patients^3,4^. In addition to acute events, medial wall perforation by acetabular screws can lead to chronic expanding hematoma, progressive osteolysis, and late loosening of the acetabular component many years after surgery, particularly in patients receiving anticoagulant therapy^5,6^. Furthermore, acetabular screws may cause sciatic nerve irritation because of hardware protrusion^7,8^.

To mitigate these risks, several anatomical and radiographic studies have described “safe zones” for acetabular screw placement based on the spatial relationship between the acetabulum and intrapelvic neurovascular structures^9,10^. The quadrant system proposed by Wasielewski et al. and subsequent 3-dimensional (3D) angiographic analyses have consistently identified the posterosuperior quadrant as the safest region, whereas placement in the anterosuperior and anteroinferior quadrants may pose a risk to the external iliac and obturator vessels^9-11^.

Nevertheless, the distance between intrapelvic vessels and the acetabulum varies depending on anatomical and patient-specific factors, including age, sex, laterality, and vessel morphology^12,13^. Moreover, studies using cadaveric specimens may be limited quantitatively because of postmortem tissue shrinkage, dehydration, and nonphysiological positioning^14^. Therefore, a rigorous, in vivo data-based safety concept that minimizes the need to account for patient-specific variation in neurovascular anatomy is needed.

The purpose of this study was to use 3D computed tomography (CT) data to virtually simulate and map intraosseous safe zones—defined as regions where screws remain entirely within bone, without penetrating the medial wall—for acetabular cup screw placement in cementless THA.

## Materials and Methods

### Patients

In this retrospective, single-center cohort study, 47 consecutive female patients with osteonecrosis of the femoral head who underwent preoperative CT evaluation of their right hip at our institution between January 2018 and April 2025 were screened for inclusion. The exclusion criteria were applied as follows: (1) center-edge (CE) angle ≤ 25°, indicating acetabular dysplasia; (2) Association Research Circulation Osseous (ARCO) stage 4 osteonecrosis with acetabular changes^15^; and (3) a history of prior surgery. After applying these eligibility criteria, 30 patients (30 right hips) with normal acetabular morphology were included in the final analysis.

Ethical approval was obtained from the institutional research committee (approval number 2986). The study adhered to the STROBE guidelines^16^.

### CT Acquisition and 3D Modeling

CT imaging was performed using a multidetector scanner with a slice thickness of 1.0 mm and voxel size of 0.625 mm. Three-dimensional reconstructions were generated using SYNAPSE VINCENT (FUJIFILM Medical Systems, Tokyo, Japan). To standardize pelvic orientation, the functional pelvic plane (FPP) was defined as the coronal plane passing through both anterior superior iliac spines (ASIS) and maintained parallel to the floor^17,18^. All subsequent measurements were referenced to the FPP.

A sphere was fitted to the acetabular rim to determine the acetabular center, and a hemispherical cup model was virtually positioned such that its center coincided with this acetabular center. The cup was oriented at a radiographic inclination of 40° and a radiographic anteversion of 15° relative to the FPP, in accordance with the standard established by Lewinnek et al^19^.

### Measurement Planes

The reference axis (12 o’clock direction) was defined as the vector from the cup center toward the ipsilateral ASIS. The acetabular cup was conceptualized as a clock face, with directions measured clockwise from the reference axis. Multiplanar reconstruction images perpendicular to the cup rim plane were generated clockwise at 15° intervals, yielding 12 equally spaced cross-sectional planes. Each plane provided 2 opposing directions (180° apart), yielding a total of 24 measurement directions (Fig. 1).

**Fig. 1:**
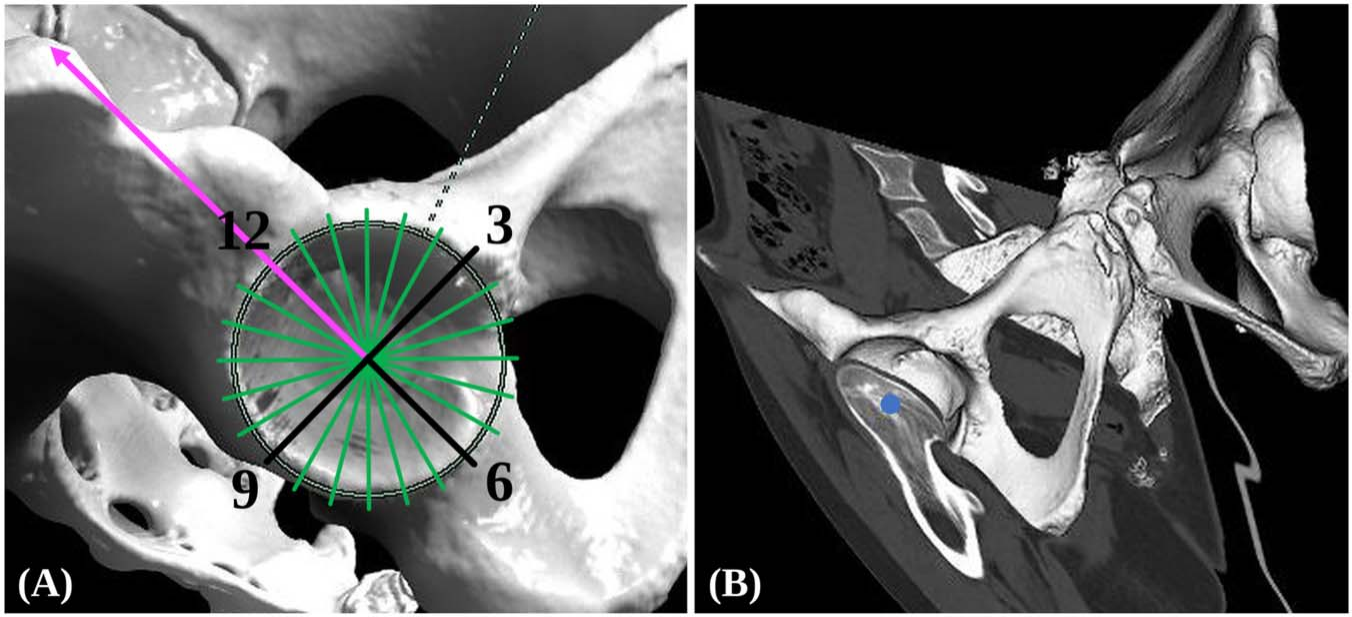
Twelve cross-sectional planes of the acetabulum. **Fig. 1-A** Three-dimensional CT reconstruction. The acetabulum is divided into 12 equally spaced cross-sectional planes centered on the cup (green lines). The reference axis is defined by the line connecting the ASIS and the cup center (pink line). **Fig. 1-B** Representative three-dimensional CT reconstruction with a multiplanar reconstruction section along the 12 to 6 o’clock plane passing through the ASIS. ASIS = anterior superior iliac spine, and CT = computed tomography.

### Intraosseous Safe Zone Definition

Screw insertion was assumed to originate from the cup center^10^. Six screw lengths (15, 20, 25, 30, 40, and 50 mm) were evaluated in each of the 24 directions. In each cross-sectional image, the distance (D) from the cup center to the medial cortex was measured along the screw trajectory. The available bone depth was calculated as D minus the cup radius (r). A screw was considered intraosseous when its length did not exceed this value (D−r). For each screw length, using the cup center as the reference point, the insertion angle at which the screw remained intraosseous without breaching the medial cortex was calculated as the angle between the cup rim plane and the screw axis (Fig. 2-A). The angular range in which each screw length remained entirely intraosseous was defined as the intraosseous safe zone, described using the clock-face coordinate system (Fig. 2-B).

**Fig. 2:**
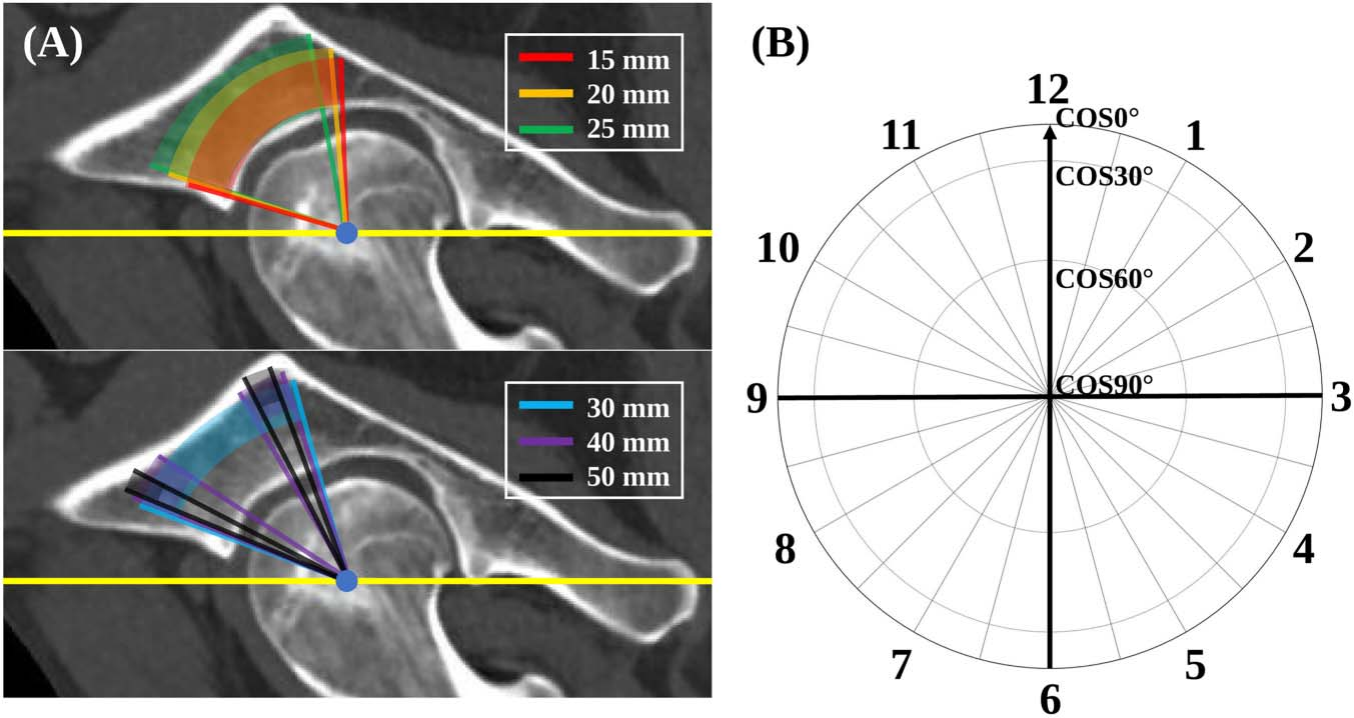
Measurement of the intraosseous safe zone and the clock-face coordinate system. **Fig. 2-A** Representative cross-sectional image of the acetabulum along the 12 to 6 o’clock plane. The yellow horizontal line passing through the femoral head center represents the plane of the acetabular cup rim, from which the screw insertion angles were measured. The colored sectors indicate the intraosseous regions permitting the insertion of screws of different lengths: red, 15 mm; orange, 20 mm; green, 25 mm; light blue, 30 mm; purple, 40 mm; and black, 50 mm. These angular measurements were obtained in 24 directions across 12 cross-sectional planes. **Fig. 2-B** Clock-face coordinate system. The acetabulum, viewed at 40° of abduction and 15° of anteversion, is represented as a unit circle. The radial coordinate is defined as |cos θ|, where θ represents the angular direction of screw insertion.

### Safe Zone Mapping and Heatmap Generation

Heatmaps were generated using Python (Matplotlib library). For each patient, the minimum and maximum safe-zone angular boundaries from the 12 cross-sectional planes were projected onto a unit circle using r = |cos θ|. In this projection, the minimum safe-zone angle was mapped to a more peripheral location (farther from the center of the clock-face coordinate system), whereas the maximum angle was mapped to a more central location, thus defining the respective boundaries for each cross section. The region bounded by 4 coordinates—defined by the minimum and maximum safe-zone angles of 2 adjacent cross sections (e.g., the 15° and 30° planes)—was treated as a continuous safe-zone region, and a value of 1 was assigned to each pixel within this region. This procedure was repeated for all planes in each of the 30 hips, producing heatmaps in which darker shading indicated a greater number of hips with intraosseous screw placement at that location.

Using the same analytical environment, the region in which all 30 hips showed intraosseous screw placement was extracted as the intraosseous safe zone. Morphological opening (SciPy library) was employed to eliminate artifactual isolated noise regions, and the resultant safe-zone contours for each screw length were overlaid on a unified clock-face diagram. Within this region, the range of insertion angles at each clock-face position, in which the screw remained intraosseous, was calculated as the difference between the maximum and minimum permissible insertion angles.

### Statistical Analysis and Reliability

Differences in intraosseous safe zones among screw lengths were compared using pairwise Wilcoxon signed-rank tests with Bonferroni correction. To assess interrater reliability, the angular boundaries of the safe zone were measured independently by 2 observers (T.S. and R.H.), who were blinded to each other’s results. Reliability was quantified using the 2-way random-effects intraclass correlation coefficient ICC (2,1), with values interpreted as excellent (>0.90), good (0.75-0.90), moderate (0.50-0.75), or poor (<0.50)^20^. All statistical analyses were performed using EZR v. 1.64 (Saitama Medical Center, Jichi Medical University), a graphical user interface for R v. 4.2.3 (R Foundation for Statistical Computing)^21^.

## Results

The median age of the patients was 50.1 years (range, 17-79 years), median height was 157.4 cm (range, 145.0-166.0 cm), median weight was 59.0 kg (range, 38.1-82.4 kg), and median CE angle was 32.3° (range, 25.2°-43.4°). According to the ARCO classification, stage 1 osteonecrosis was observed in 18 hips, stage 2 in 7 hips, stage 3A in 3 hips, and stage 3B in 2 hips. The median outer acetabular diameter was 48.8 mm (range, 45.2-51.2 mm), while the median femoral head diameter was 44.6 mm (range, 40.6-48.8 mm). The median acetabular anteversion, measured on axial CT at the level of the femoral head center, was 19.6° (range, 11.3°-31.6°), and no hip showed acetabular retroversion.

Across all screw lengths, heatmaps demonstrated the highest density in the posterosuperior region (Fig. 3). The density of the safe region decreased both superiorly and inferiorly with increasing screw length, progressively converging toward the 10 to 11 o’clock region. Limited density was observed around the 3 o’clock position on the heatmaps for 15 mm and 20 mm screws, and the intraosseous safe zone was nearly absent across the broader anterior arc.

**Fig. 3:**
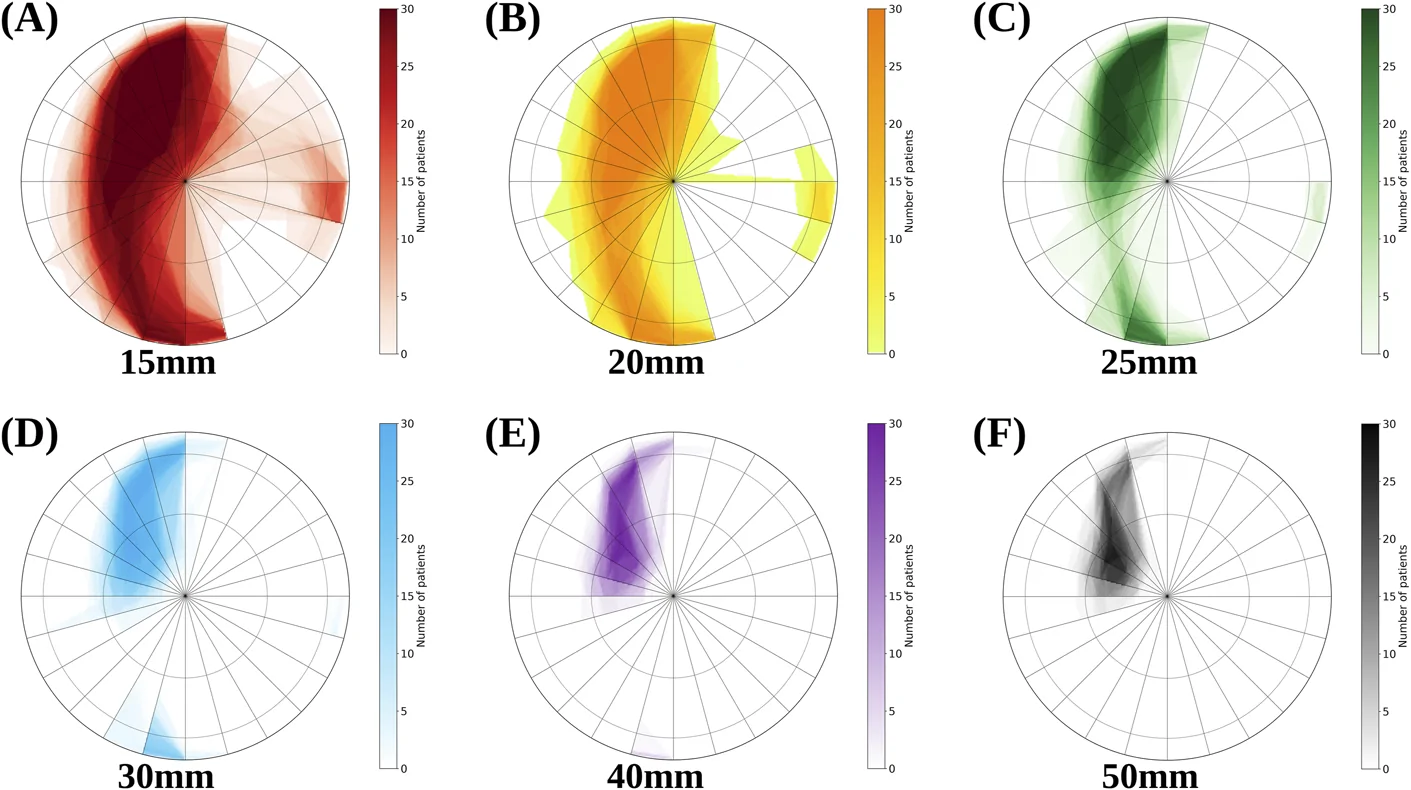
Heatmaps of intraosseous safe zones stratified by screw length (**Fig. 3-A**) 15 mm screws, (**Fig. 3-B**) 20 mm screws, (**Fig. 3-C**) 25 mm screws, (**Fig. 3-D**) 30 mm screws, (**Fig. 3-E**) 40 mm screws, and (**Fig. 3-F**) 50 mm screws. Darker shading indicates a greater number of hips in which screw placement remained entirely intraosseous at each location.

The size of the intraosseous safe zone in all hips decreased significantly in the following order: 15 mm > 20 mm > 25 mm > 30 mm (p < 0.01) (Fig. 4). As screw length decreased, the common intraosseous safe zone progressively expanded toward the posteroinferior region. Although intraosseous regions were identified in some hips for the 40 and 50 mm screws (Fig. 3), no safe region was common to all 30 hips. Therefore, no contours for these screw lengths are shown in Fig. 4. At the 70%, 80%, and 90% thresholds, the safe zones progressively converged to the 10 to 11 o’clock region for 40 mm screws and the 10 to 10:30 o’clock region for 50 mm screws (Supplementary Fig. 1).

**Fig. 4:**
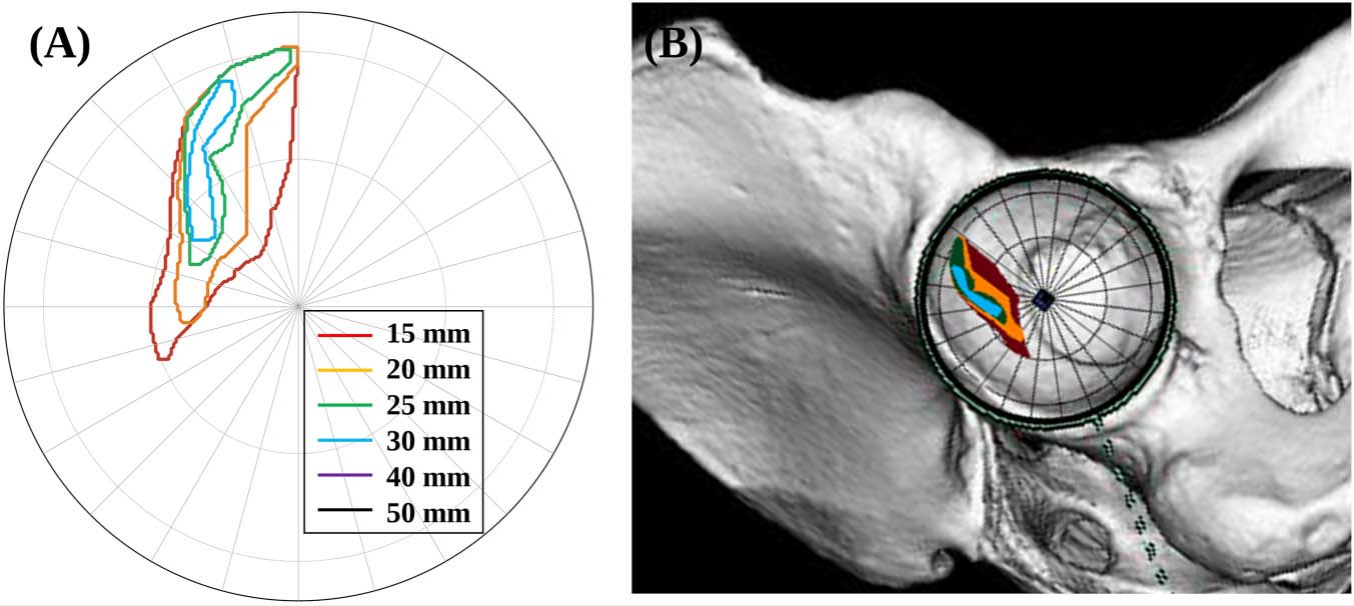
Common intraosseous safe zones shared by all 30 hips. **Fig. 4-A** Intraosseous safe zones for each screw length (15, 20, 25, and 30 mm) overlaid on a clock-face diagram. The enclosed regions indicate the angular ranges in which the screw remained entirely intraosseous, without medial wall penetration, in all 30 hips. For 40 and 50 mm screws, no safe zone in which the screw remained entirely intraosseous was identified across all 30 hips; therefore, no contours are shown for these screw lengths. **Fig. 4-B** The common intraosseous safe zones projected onto the 3-dimensional acetabular cup surface at 40° of inclination and 15° of anteversion.

The clock-face–specific angle ranges for each screw length within this intraosseous safe zone in all hips are summarized in Table 1. Across screw lengths from 15 to 25 mm, the angular ranges of the safe zone were generally the greatest at the 11 and 11:30 o’clock positions.

**Table 1:** Angular Ranges of Screw Trajectories within the Intraosseous Safe Zone in All Hips

Clock-Face Direction	Screw Length			
	15 mm	20 mm	25 mm	30 mm
8:30	5° (60.0°–65.0°)	-	-	-
9:00	12° (60.0°–72.0°)	6° (66.0°–72.0°)	-	-
9:30	12° (62.0°–74.0°)	7° (65.0°–72.0°)	-	-
10:00	16° (60.0°–76.0°)	9° (63.0°–72.0°)	5° (65.0°–70.0°)	-
10:30	24° (53.0°–77.0°)	18° (55.0°–73.0°)	12° (57.0°–69.0°)	9° (58.0°–67.0°)
11:00	36° (41.0°–77.0°)	29° (41.0°–70.0°)	12° (42.0°–54.0°)	5° (46.0°–51.0°)
11:30	38° (32.0°–70.0°)	18° (32.0°–50.0°)	13° (32.0°–45.0°)	-
12:00 (ASIS)	12° (26.0°–38.0°)	9° (26.0°–35.0°)	-	-

ASIS = anterior superior iliac spine.

Values represent the angular range (minimum to maximum angle) within which intraosseous containment was achieved for all 30 hips. Hyphens (“-”) indicate the absence of a safe zone in that direction for the specified screw length.

The ICCs for the interrater reliability of the safe-zone angular boundary measurements were 0.942 for 15 mm screws, 0.938 for 20 mm screws, 0.915 for 25 mm screws, 0.924 for 30 mm screws, 0.912 for 40 mm screws, and 0.906 for 50 mm screws. All values were considered excellent.

## Discussion

In this study, 3D CT-based multiplanar reconstruction was used to measure acetabular bone thickness and simulate intraosseous safe zones for acetabular cup screw placement. Notably, the heatmap generated for 25 mm screws closely approximated the posterosuperior safe quadrant described by Wasielewski et al. in their landmark cadaveric study^9^, lending validity to our simulation methodology. Although the intraosseous safe zone in all hips was narrower than the conventional posterosuperior safe quadrant^9^, the present study demonstrated progressive contraction of the safe zone with increasing screw length, converging toward a narrow posterosuperior corridor around the 10 to 11 o’clock region. As screw length increased beyond 30 mm, no universally shared safe zone could be identified, whereas the 11 to 11:30 o’clock region consistently demonstrated the widest angular margin and remained the most robust osseous corridor across 15 to 25 mm screw lengths.

The heatmap of the intraosseous safe zone showed the highest density in the posterosuperior region, consistent with previous studies, indicating that the posterosuperior acetabulum is the safest region for screw placement^9,10^. The posterosuperior acetabulum has greater bone thickness than other regions^22,23^, and biomechanical studies have shown that, in unicortical fixation, pull-out strength is the greatest in the superior region and is not correlated with screw length^24^. Although some surgeons favor bicortical screw fixation, recent evidence suggests that unicortical fixation is sufficient in primary THA, with no difference in implant survival or loosening between cases with and without screws^25,26^. Collectively, these findings suggest that directing unicortical screws toward the posterosuperior region alone is sufficient for primary THA.

Revision THA frequently requires ancillary screw fixation because of compromised bone stock and inadequate press-fit fixation^27^. In such situations, surgeons may use longer screws or direct screws toward the ischium or pubis to achieve fixation in the remaining host bone. In the present study, shortening the screw length to 20 or 15 mm expanded the common intraosseous safe zone toward the posteroinferior region. Consistent with previous reports demonstrating safe ischial screw placement^11^ and improved stability with posteroinferior fixation^28,29^, these findings suggest that short posteroinferior screws may provide both intraosseous safety and biomechanical benefit. Although no common safe zone was identified for 40 or 50 mm screws, the remaining osseous corridor converged toward the 10 to 10:30 o’clock region, providing a useful anatomical reference when longer screw fixation is considered.

Recent advances in computer-assisted orthopedic surgery, including navigation and robotic-assisted THA, facilitate patient-specific preoperative planning and, in combination with contrast-enhanced CT, visualize the intrapelvic vascular anatomy. Although these technologies enable individualized risk assessment, they require additional imaging, costs, and specialized equipment^30^. The intraosseous safe zone proposed in this study provides a simple, broadly applicable framework based solely on osseous anatomy, without requiring contrast imaging. These approaches may be complementary, with intraosseous mapping offering a bone-based safety baseline and computer-assisted orthopedic surgery adding patient-specific refinement.

This study had several limitations. First, the sample size of 30 hips limited the representativeness of the safe-zone maps. Second, the study was restricted to Japanese female patients with osteonecrosis of the femoral head and normal acetabular morphology. Therefore, the intraosseous safe zone in larger patients, male patients, and other racial or ethnic populations may be substantially altered, and the present results cannot be extrapolated to these groups without dedicated validation. Third, the cup was virtually positioned at a fixed orientation according to the Lewinnek target. In practice, cup orientation varies with the surgical approach, pelvic tilt, anatomy, and intraoperative constraints; accordingly, a different orientation would shift the intraosseous safe zone, and this variability was not modeled. Fourth, this virtual simulation did not account for acetabular wall thinning caused by reaming, which should be considered during preoperative planning. Fifth, screws were assumed to be inserted from the cup center, whereas for real implants, the trajectory is constrained by the screw hole number, position, and orientation in a specific acetabular shell^31^. Despite these limitations, the high interrater reliability confirms the reproducibility of this approach. Future multicenter studies incorporating diverse patient populations, variable cup orientations, postreaming anatomy, implant-specific screw-hole constraints, and acetabular bone defects encountered in revision THA are warranted to validate and refine the safe-zone maps.

In conclusion, this study defines the intraosseous safe zone for acetabular screw placement based on 3D CT-derived bone thickness, offering a practical framework for preoperative planning and conceptualizing safer screw trajectories in THA.

## Funding

This work was supported by JSPS KAKENHI Grant Number JP25K19959.

## Appendix

Supporting material provided by the authors is posted with the online version of this article as a data supplement at jbjs.org (https://links.lww.com/JBJSOA/B391). This content was not copyedited or verified by JBJS.
